# Genomic comparison of the temperate coral *Astrangia poculata* with tropical corals yields insights into winter quiescence, innate immunity, and sexual reproduction

**DOI:** 10.1093/g3journal/jkaf033

**Published:** 2025-02-18

**Authors:** Kathryn H Stankiewicz, Nadège Guiglielmoni, Sheila A Kitchen, Jean-François Flot, Katie L Barott, Sarah W Davies, John R Finnerty, Sean P Grace, Leslie S Kaufman, Hollie M Putnam, Randi D Rotjan, Koty H Sharp, Esther C Peters, Iliana B Baums

**Affiliations:** Department of Biology, The Pennsylvania State University, University Park, PA 16802, USA; Institute for Systems Biology, Seattle, WA 98109, USA; Department of Marine Biology, Université libre de Bruxelles (ULB), Brussels 1050, Belgium; Department of Biology, The Pennsylvania State University, University Park, PA 16802, USA; Department of Marine Biology, Texas A&M University at Galveston, Galveston, TX 77554, USA; Department of Marine Biology, Université libre de Bruxelles (ULB), Brussels 1050, Belgium; Interuniversity Institute of Bioinformatics in Brussels —(IB), Brussels 1050, Belgium; Department of Biology, University of Pennsylvania, Philadelphia, PA 19104, USA; Department of Biology, Boston University, Boston, MA 02215, USA; Department of Biology, Boston University, Boston, MA 02215, USA; Department of Biology & Werth Center for Coastal and Marine Studies, Southern Connecticut State University, New Haven, CT 06515, USA; Department of Biology, Boston University, Boston, MA 02215, USA; Department of Biological Sciences, University of Rhode Island, Kingston, RI 02881, USA; Department of Biology, Boston University, Boston, MA 02215, USA; Department of Biology, Marine Biology, and Environmental Science, Roger Williams University, Bristol, RI 02809, USA; Department of Environmental Science and Policy, George Mason University, Fairfax, VA 22030, USA; Department of Biology, The Pennsylvania State University, University Park, PA 16802, USA; Helmholtz Institute for Functional Marine Biodiversity at the University of Oldenburg (HIFMB), Carl von Ossietzky Universität Oldenburg, Oldenburg 26129, Germany; Alfred Wegener Institute, Helmholtz-Centre for Polar and Marine Research, Bremerhaven 27570, Germany; Institute for Chemistry and Biology of the Marine Environment (ICBM), School of Mathematics and Science, Carl von Ossietzky Universität Oldenburg, Oldenburg 26129, Germany

**Keywords:** chromosome-level genome assembly, corals, scleractinian, facultative symbiosis, evolution, gene family expansion, whole-genome duplication, dormancy, mass spawning

## Abstract

Facultatively symbiotic corals provide important experimental models to explore the establishment, maintenance, and breakdown of the mutualism between corals and members of the algal family Symbiodiniaceae. Here, we report the de novo chromosome-scale genome assembly and annotation of the facultatively symbiotic, temperate coral *Astrangia poculata*. Though widespread segmental/tandem duplications of genomic regions were detected, we did not find strong evidence of a whole-genome duplication event. Comparison of the gene arrangement between *As. poculata* and the tropical coral *Acropora millepora* revealed considerable conserved colinearity despite ∼415 million years of divergence. Gene families related to sperm hyperactivation and innate immunity, including lectins, were found to contain more genes in *Ac. millepora* relative to *As. poculata*. Sperm hyperactivation in *Ac. millepora* is expected given the extreme requirements of gamete competition during mass spawning events in tropical corals, while lectins are important in the establishment of coral–algal symbiosis. By contrast, gene families involved in sleep promotion, feeding suppression, and circadian sleep/wake cycle processes were expanded in *As. poculata*. These expanded gene families may play a role in *As. poculata*'s ability to enter a dormancy-like state (winter quiescence) to survive freezing temperatures at the northern edges of the species' range.

## Introduction

Anthozoa, the largest class within the phylum Cnidaria, includes some of the most ecologically important and oldest clades of marine metazoans, estimated to have evolved as early as 771 million years ago (Mya) (McFadden *et al*. 2021). Among these are corals, a diverse group of solitary and colonial organisms that can form a symbiotic association with algae of the family Symbiodiniaceae in the shallow water of the tropic and temperate zones (LaJeunesse *et al*. 2018). Stony corals of the order Scleractinia contain the engineers of reef ecosystems and are generally divided into 2 major clades, Complexa and Robusta, which diverged ∼415 Mya (Romano and Palumbi 1996; Kitahara *et al.* 2010; Stolarski *et al*. 2011). Over the past several decades, tropical coral species have undergone mass mortality due to their sensitivity to bleaching in the face of anthropogenic climate change (Bellwood *et al.* 2004; DeCarlo *et al*. 2017; Hughes *et al*. 2017). Understanding differences between species of variable temperature tolerance and adaptive strategies has become a focus for conservation efforts. In the “robust” clade, the temperate coral *Astrangia poculata* (the Northern Star Coral) has recently emerged as a model system for these comparisons due to its facultative symbiosis and temperature tolerance ranging from near freezing to 24°C in Narragansett Bay, the northern part of its distribution (Jacques *et al.* 1983).

**Fig. 1.**
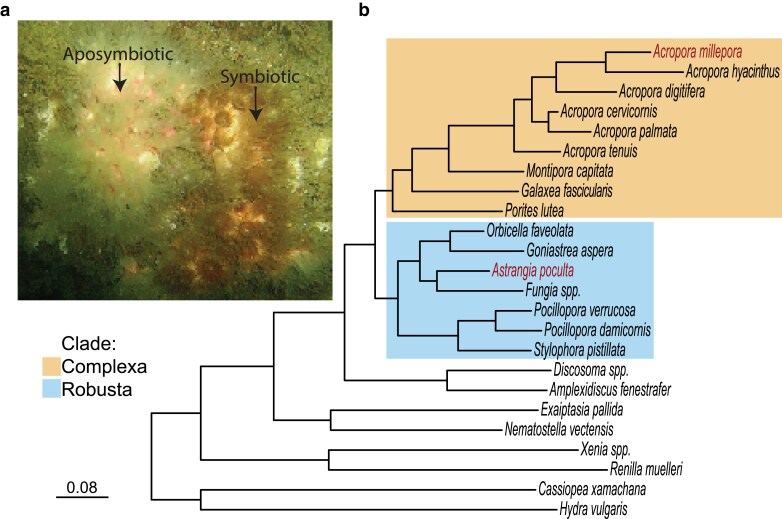
Photograph of *As. poculata* and phylogenetic tree. a) Underwater photograph of *As. poculata* colony with extended tentacles exhibiting aposymbiotic (white appearance, left) and symbiotic (brown appearance, right) states. Photograph credit to Sean P. Grace. b) Phylogeny of cnidarians included in the comparative genomic analyses of this study. Scleractinian coral clades are highlighted (top box, yellow = Complexa; bottom box, blue = Robusta). *Astrangia poculata* and *Ac. millepora* are highlighted in red. The species tree was inferred using the STAG algorithm and rooted using the STRIDE algorithm. Branch lengths represent the number of substitutions per site.

*Astrangia poculata*, like many other shallow water corals, hosts a photosynthesizing algal symbiont, *Breviolum psygmophilum* (Lajeunesse *et al.* 2012). The symbiosis is facultative, with a gradient of symbiont density existing among individual polyps within a single colony of *As. poculata* and between sympatric colonies (Dimond and Carrington 2007) as revealed by an aposymbiotic “white” appearance, nearly or entirely devoid of symbionts, or a symbiotic “brown” appearance (Fig. 1a). Unlike many other shallow water coral species, *As. poculata* occurs across a broad temperature and latitudinal gradient from southern Massachusetts to the Gulf of Mexico (Cummings 1983; Peters *et al*. 1988; Dimond and Carrington 2007; Dimond *et al* 2013). At the northernmost parts of its range, during winter months high in the intertidal zone, *As. poculata* experiences a quiescence characterized by a dormancy state of reduced feeding and growth (Supplementary Fig. 1; Jacques *et al*. 1983; Grace 2017). This appears to be an adaptation of *As. poculata* to the intertidal zone, where it is vulnerable to desiccation, predation, and extreme shifts in salinity and temperature. *Astrangia poculata's* hardiness combined with the ability of researchers to experimentally isolate the contributions of the host and the symbiont in aposymbiotic and symbiotic colonies has made this species particularly interesting for the study of coral symbiosis in the face of climate change.

Previous work found signatures of adaptation in the thermal tolerance of *As. poculata* across the species' range, with a cold-adapted population exhibiting higher metabolic rates than warm-adapted populations across several temperature treatments (Aichelman *et al.* 2019). Symbiotic and aposymbiotic *As. poculata* respond differently to warm and cold temperatures at both the organismal and transcriptomic levels. Symbiotic colonies significantly outperformed aposymbiotic colonies at 9, 18, and 24°C with respect to wound healing (Burmester *et al*. 2017), while aposymbiotic colonies responded more strongly transcriptionally to a cold exposure than to a heat exposure treatment, particularly through upregulation of genes involved in the myosin complex, proteasome core, translation regulator activity, nucleic acid binding, extracellular matrix structural constituent, muscle system process, and proteolysis (Wuitchik *et al*. 2021). In a separate thermal stress experiment, the algal endosymbiont *B. psygmophilum* responded more strongly than the *As. poculata* host to chronic heat stress (Chan *et al*. 2021). Further, season, rather than symbiont state, was shown to drive the structure of the microbiome in *As. poculata* (Sharp *et al.* 2017). Thus, across multiple scales of measurement, thermal variation, particularly exposure to cold temperatures, appears to play an important role in the biology and ecology of this coral. However, the evolutionary roots and the genomic mechanisms driving the response to environmental change remain unclear for *As. poculata*, and for many other corals.

The use of comparative genomics has shed light on the evolution of basal metazoans. For example, studies have suggested the potential roles of whole-genome duplication (WGD) (Mao and Satoh 2019), horizontal gene transfer (Bhattacharya *et al*. 2016), and de novo biosynthesis pathways (Ying *et al*. 2018) in coral evolutionary trajectories. Many of these comparisons have focused on complex vs robust lineages, for which there exists a deep evolutionary split based on molecular and phylogenetic evidence (Fig. 1b; Romano and Palumbi 1996; Kitahara *et al*. 2010; Stolarski *et al*. 2011). However, it is not well understood how phylogenetically widespread these genomic traits may be, and the inclusion of other corals representing a wider array of ecological niches and adaptive strategies is warranted, which requires additional genomic resources.

While over the past several years there has been an increase in the availability of genomic resources for cnidarians, currently few of them are at chromosome-scale (however, see Fuller *et al*. 2020; Hu *et al*. 2020; Stephens *et al*. 2022; Locatelli et al. 2024) and none represent a facultatively symbiotic, temperate coral. To fill this gap, we present here the chromosome-scale assembly of a male *As. poculata* colony. The assembly is among the most contiguous and complete of available coral genome assemblies to date. We characterized the structure and content of the *As. poculata* genome to investigate potential genomic drivers underlying this species' unique temperature tolerance and flexible symbiotic state. To determine the sex of the sequenced colony, we examined histological sections from the colony. Our aims were to (1) produce a high-quality reference genome for *As. poculata*, (2) use this genome assembly to explore several potential genomic mechanisms that may contribute to the unique plasticity of the species, and (3) characterize the degree of similarity of the gene repertoire and genome organization among *As. poculata* and other corals.

## Methods

### Genome sequencing

An aposymbiotic colony was collected from Fort Wetherill State Park in Jamestown, Rhode Island, USA in October of 2017. To avoid sequencing the symbiont, a colony with a minimal density of *B. psygmophilum* in its tissue was selected based on the colony's white appearance. A subsample of this colony was collected, frozen, and sent for DNA extraction, library preparation, and sequencing at Dovetail Genomics (Scotts Valley, CA). Two subsamples from this colony were maintained in aquaria at the Pennsylvania State University, 2 at Boston University, and 2 at the University of Rhode Island for future use.

Sequencing involved a multistrategy approach and included 2 Illumina (San Diego, CA) datasets of paired-end 150 base-pair (bp) reads: one with 414 million reads and an estimated average insert size of 395 bp, and the second with 235 million reads and an estimated average insert size of 484 bp. Three Hi-C libraries were produced with 198 million, 266 million, and 257 million of 150 bp paired-end reads each. DNA extraction, library preparation, and sequencing of the Illumina and Hi-C data were carried out by Dovetail Genomics (https://dovetailgenomics.com/). All Illumina and Hi-C reads were trimmed using Cutadapt v2.9 with default settings (Martin 2011).

In preparation for Oxford Nanopore (Oxford, UK) sequencing, extracted genomic DNA was purified with AMPure XP Beads to improve the purity. Short fragments were discarded using Circulomics Short Reads Eliminator XS (Circulomics, Pacific Biosciences). The library was prepared using the Nanopore Ligation Sequencing Kit LSK109, starting with 2.1 μg of DNA, and yielded 1.4 μg of library. The final library was sequenced with a MinION on an R9.4 flow cell with fast base calling (Jain *et al.* 2016). The flow cell was washed and reloaded 3 times (281 ng of DNA for the first load, 187 ng for subsequent loads). A total output of 6.79 Gigabases (Gb) was obtained with an N_50_ of 18 kilobases (kb) and an N_90_ of 5 kb. The reads were trimmed of the adaptors with Porechop (available at https://github.com/rrwick/Porechop), using default parameters. After trimming, the dataset reached 6.77 Gb.

### De novo genome assembly

Several assembly strategies were compared to select the most robust approach (Supplementary Table 1). Assemblers that were investigated include Raven (Vaser and Šikić 2019), Flye (Kolmogorov *et al.* 2019), Canu (Koren *et al*. 2017), and wtdbg2 (Ruan and Li 2020). Ultimately, the genome was assembled using wtdbg2 v2.5 under default settings. Haplotigs were purged using purge_haplotigs v1.1.1 (Roach *et al.* 2018) with default settings [following the recommendations in Guiglielmoni *et al*. (2021)] with both Illumina datasets mapped using bowtie2 v2.3.5.1 (Langmead and Salzberg 2012). Polishing was done using HyPo v1.0.3 (Kundu *et al.* 2019). Hi-C reads were mapped to the assembly and processed using bowtie2 v2.3.5.1 and hicstuff v2.3.0 (Matthey-Doret *et al*. 2020) with the parameters --enzyme DpnII --iterative --aligner bowtie2. The draft assembly was then scaffolded using instaGRAAL v0.1.6 no-opengl branch (Baudry *et al*. 2020), with default parameters (--levels 4, --cycles 100, --coverage-std 1, --neighborhood 5). The output was then refined using the module instaGRAAL-polish. BUSCO v5.4.7 was used to assess the completeness of the assembly (Simão *et al.* 2015) against the Metazoa odb10 lineage. The genome size was estimated with the Illumina dataset of 235 million reads and the module kmercount.sh from BBmap v38.79 (Bushnell 2014). The circularized mitochondrial genome was assembled from the Illumina reads using NOVOPlasty v2.7.2 (Dierckxsens *et al.* 2016) with the publicly available *Acropora digitifera* mitochondrial genome as a reference (GenBank: KF448535.1). Our 14 chromosome-scale scaffolds were named Ap1, Ap2, …, Ap14 on the basis of their homologies with *Acropora millepora* chromosome-scale scaffolds bearing the same number (in the absence of a published karyotype for *As. poculata*).

### Transcriptome assembly

RNA sequencing data from Chan *et al*. (2021) were used to construct a de novo transcriptome. First, to limit the potential for symbiont contamination, only reads for aposymbiotic individuals were used for the host transcriptome assembly. Reads were trimmed using Cutadapt v3.4 (Martin 2011) with a quality cutoff of 15 and a minimum read length of 50 nucleotides. The trimmed reads were then assembled into transcripts using rnaSPades v3.12.0 (Bushmanova *et al.* 2019). To further reduce the possibility of contamination from the algal endosymbiont, a custom database of Symbiodiniaceae protein sequences was assembled that included the following species: *Cladocopium goreaui* (Liu *et al.* 2018b), *B. psygmophilum* (Parkinson *et al*. 2016), *Breviolum minutum* (Shoguchi *et al*. 2013), *Fugacium kawaguti* (Liu *et al.* 2018a, 2018b), *Symbiodinium fitti* (Reich *et al*. 2021), *Symbiodinium microadriaticum* (Aranda *et al*. 2016), and *Symbiodinium tridacnidorum* (González-Pech *et al*. 2021). A BLAST nucleotide-to-protein alignment (blastx) of the assembled *As. poculata* transcriptome was conducted against this database using BLAST v2.6.0 (Altschul *et al.* 1990; Camacho *et al*. 2009). Transcripts with at least 80% identity and with at least 100 bp mapped length were filtered from the transcriptome.

### Genome annotation

To annotate repetitive content, de novo transposable element family identification and modeling were conducted using RepeatModeler v1.0.1 (Smit and Hubley 2008). RepeatMasker v4.0.7 (Smit *et al.* 2015) was then used to soft-mask repetitive regions prior to gene modeling. Subsequent gene prediction included a multitool approach. First, ab initio gene prediction was done using GeneMark-ES v3.51 (Lukashin and Borodovsky 1998). Gene prediction using protein-based evidence was conducted with Exonerate v2.4.0 (Slater and Birney 2005) using the UniProt eukaryote database (downloaded 2017 December 28). RNA-seq reads from Chan *et al*. (2021) were aligned to the genome using STAR v020201 (Dobin *et al*. 2013) and incorporated into the automated training of gene prediction using Braker2 v2.1.2 (Brůna et al. 2021). From the resulting predictions, high-quality gene models (HiQ), here defined as those having ≥90% RNA-seq coverage support, were extracted. Gene prediction informed by transcriptomic evidence was carried out using PASA v2.3.3 (Haas *et al*. 2003) with the flag --TRANSDECODER to keep only the longest open reading frame per group of transcript isoforms (see section “*Transcriptome assembly*” above for transcriptome assembly details). Consensus gene predictions were acquired using EvidenceModeler v1.1.1 (Haas *et al*. 2008) weighting each line of evidence as follows: ab initio predictions: 1; RNA-seq based evidence: 1; protein-based evidence: 1; HiQ: 5; and transcriptomic-based evidence: 10. PASA v2.3.3 was run a second time to update gene models and add annotations of untranslated regions (UTRs) using the consensus gene prediction from EVidenceModeler. Finally, tRNAscan-Se v1.3.1 (Chan and Lowe 2019) was used to annotate transfer RNAs. Genome Annotation Generator (GAG) v2.0.1 (Geib *et al*. 2018) was used to extract coding sequence (CDS), protein, and mRNA sequences.

Functional annotation of predicted genes was conducted based on sequence similarity using BLAST v2.6.0 (Altschul *et al*. 1990; Camacho *et al*. 2009) searches via blastp with the settings of eval = 1e−5, max target seqs = 5, and max hsp = 1. Blast queries were conducted against 3 databases: NCBI nr, UniProt Swiss-Prot, and TrEMBL. Hits to each database were combined and annotated with Gene Ontology (GO) terms using the UniProt-GOA mapping.

### Investigating the possibility of whole-genome duplication

Given the recent suggestion of a possible WGD event in the genus *Acropora* (Mao and Satoh 2019), we set out to determine whether a similar event may have occurred in *As. poculata*. Detection and classification of duplication in the genome was carried out in several ways. First, estimates were conducted using the tool MCScanX under default settings (Wang *et al*. 2012). Results were then compared to a run under more relaxed settings (max gap size increased to 50). In both cases, duplication origins were classified using the duplicate_gene_classifier module.

A second method of WGD detection employed was the tool wgd v1.1.2 (Zwaenepoel and Van de Peer 2018), which relies on the distributions of synonymous substitutions per synonymous site (*Ks*). To conduct the analysis, genome-wide CDSs were filtered for the longest translatable isoform of each gene. wgd v1.1.2 was then run using the diamond aligner to compute the whole-paranome (the collection of all duplicate genes in a genome). A *Ks* distribution was constructed in pairwise mode and kernel density estimates were subsequently fit to the distribution and visualized. A *Ks* distribution for anchor pairs, defined as paralogs located on colinear duplicated segments, was similarly constructed and visualized. The shape of the *Ks* distribution was inspected for detection of ancient WGD with the expectation of an exponential decay shape in the absence of a WGD event (Zwaenepoel and Van de Peer 2018).

Lastly, the whole-genome synteny aligner Satsuma2 (available at https://github.com/bioinfologics/satsuma2) was used to detect microhomologous regions between *As. poculata* chromosomes. Syntenic blocks of homologous sequences arranged in a colinear fashion between chromosomes were then plotted using the tool Orthodotter (https://github.com/institut-de-genomique/orthodotter) to produce an Oxford grid (Edwards 1991), an approach used previously to detect WGD in arthropods (Schwager *et al*. 2017).

### Comparative genomics: gene family and conserved synteny analysis

To characterize the genome organization and content of *As. poculata* relative to other cnidarians, we completed several comparative genomic analyses. Phylogenetic orthology inferences were carried out using OrthoFinder2 v2.4.0 (Emms and Kelly 2019) on *As. poculata* and 23 other available cnidarian proteomes (Fig. 1b; Supplementary Table 2) using default parameters with the species tree inferred using the STAG algorithm (Emms and Kelly 2018) and rooted using the STRIDE algorithm (Emms and Kelly 2017). The phylogenetic species tree was visualized using the R package “ggtree” v3.6.2 (Xu et al. 2022 ). GO enrichment of gene families unique to *As. poculata* was conducted using the clusterProfiler package (Yu *et al.* 2012) implemented in R v4.0.5 with a *P*-value cutoff of 0.05, a multiple testing correction method of Benjamini–Hochberg procedure (Benjamini and Hochberg 1995), and a *q*-value cutoff of 0.2.

*Acropora millepora*, an obligately symbiotic coral of the complex clade, was selected for more detailed comparison with *As. poculata* as its assembly was similarly complete. The sizes of gene families common to the 2 species were compared following the methods of González-Pech *et al*. (2021). Using the orthogroups previously identified by Orthofinder2, size differences of gene families shared between *As. poculata* and *Ac. millepora* were evaluated using Fisher's exact test with the multiple testing correction method of Benjamini–Hochberg (Benjamini and Hochberg 1995) and a significance threshold of adjusted *P* ≤ 0.05. GO enrichment of gene families significantly larger in *As. poculata* and in *Ac. millepora* was conducted separately using the same methods as described above.

Conserved synteny between *As. poculata* and *Ac. millepora* was assessed with MCScanX_h (Wang *et al*. 2012) using the homologous genes between the species identified by OrthoFinder2. Default parameters were used to identify colinear blocks (gap size of 25 genes allowable, minimum of 5 genes per colinear block).

### Histological examination

Three small fragments from one of the 2 still-living fragments of the sequenced *As. poculata* colony that were being maintained at Boston University were placed in Z-Fix concentrate:seawater (1:4) fixative in November 2017 and processed in the Histology Laboratory at George Mason University (GMU). Samples were decalcified in 10% ethylenediaminetetraacetic acid (EDTA) pH 7, trimmed into 5 mm strips and placed in labeled cassettes, dehydrated in a series of ethanol solutions (70–100%), cleared using Clearify (StatLab), and embedded in Paraplast Regular (Leica Microsystems Surgipath). Tissue sections, 4 µm thick, were mounted on clean glass microscope slides and stained with Harris's hematoxylin (Statlab) and eosin–phloxine and Giemsa (StatLab) procedures (Price and Peters 2018), then covered with Permount (Fisher Scientific) and a glass cover slip and examined using light microscopy.

## Results

### Genome statistics and assembly quality

**Fig. 2.**
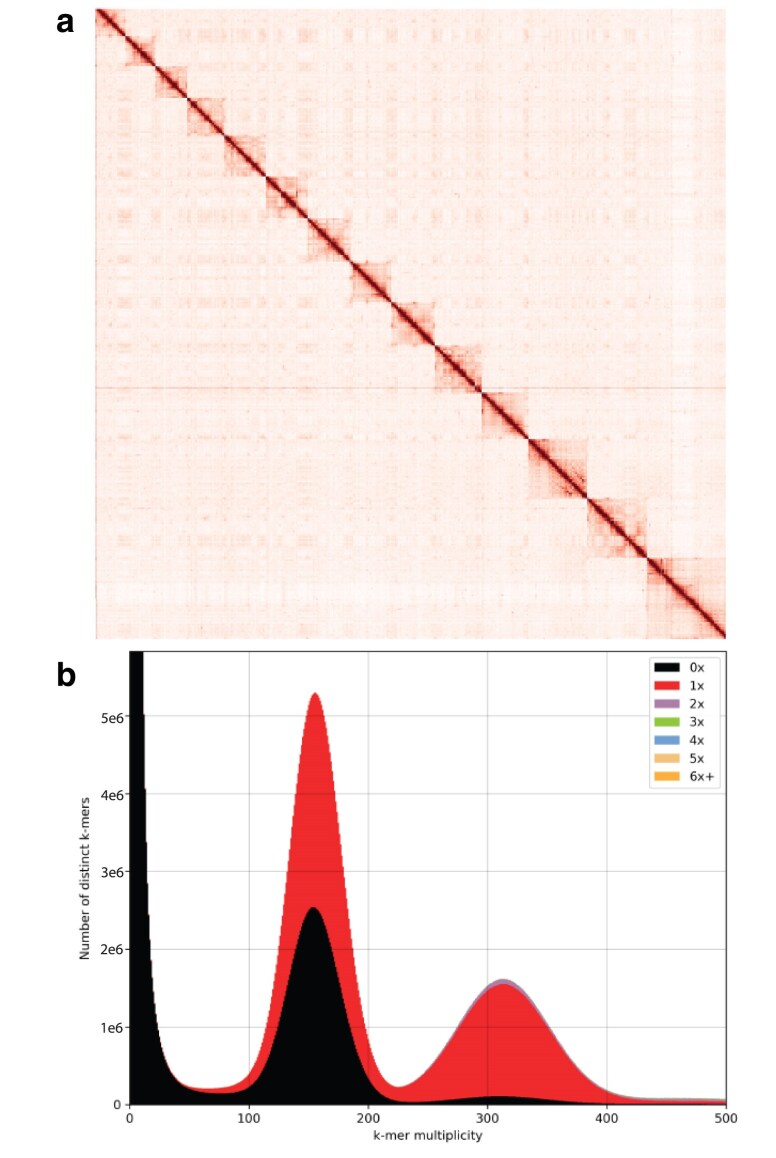
*Astrangia poculata* genome assembly. a) Proximity ligation sequencing data (Hi-C) contact map displaying the 14 chromosome-level scaffolds of the *As. poculata* assembly. Interaction points between chromosomes are represented by red dots with binning = 100 kb. Chromosomes are ordered by size from smallest to largest. b) K-mer Analysis Toolkit (KAT) plot (Mapleson *et al*. 2017) of distinct *k*-mer duplicity and the number of times these *k-*mers are represented in the final *As. poculata* genome assembly, with *k* = 27.

Of the 4 assemblers tried, wtdbg2 (Ruan and Li, 2020) produced the highest quality assembly (Fig. 2; Supplementary Table 1). After purging, polishing, and scaffolding, the assembly using wtdbg2 had an N_50_ of 31 Mb and a BUSCO score of 95.5% (93.8% single copies, 1.7% duplicate copies, 2.1% fragmented, and 2.4% missing; Table 1). The assembly size of 458 Mb is in line with BBMap predicted haploid size of 453 Mb (estimated ploidy of 2 and 40.95% repetitive). The 14 chromosome-level scaffolds match the known 2*n* = 28 formula typical of most scleractinian corals (Fig. 2a; Supplementary Table 3; Flot *et al*. 2006). Further, the *k*-mer completeness was 52.24%, close to the expected 50% for a haploid assembly (Fig. 2b). Gene prediction of the *As. poculata* genome assembly yielded 44,839 gene models, of which 1,613 had functional annotations to transposons, leaving 38,998 protein-coding genes excluding repetitive elements (Table 1). RepeatMasker predicted 39.29% of the genomic bases as repetitive elements, with an estimated GC content of 38.50%. Additionally, tRNAscan-Se identified 6,951 transfer RNAs. The assembled transcriptome (used to assist in gene modeling), included 516,681 assembled transcripts and a BUSCO score of 90.8% (Single: 32.3%, Duplicated: 58.5%, Fragmented: 6.4%, Missing: 2.8%). The high level of duplicates in the transcriptome results from retaining all isoforms for every gene.

**Table 1.** Assembly summary statistics for the *As. poculata* genome.

Metric	Value
Assembly size (Mb)	458
Number of contigs	488
N50 (Mb)	31
Genome BUSCO (%) [singles; duplicates; missing; fragmented]	95.5 (93.8; 1.7; 2.1; 2.4)
k-mer completeness (%)	52.24
Number of genes	44,839
Gene density (genes/Mb)	97.9
Average gene length (bp)	5,204
Average exon length (bp)	244
Average intron length (bp)	1,071
Average CDS length (bp)	1,268
Gene model BUSCO (%) [singles; duplicates; missing; fragmented]	92.9 (87.1; 5.8; 2.7; 4.4)

### Whole-genome duplication

**Fig. 3.**
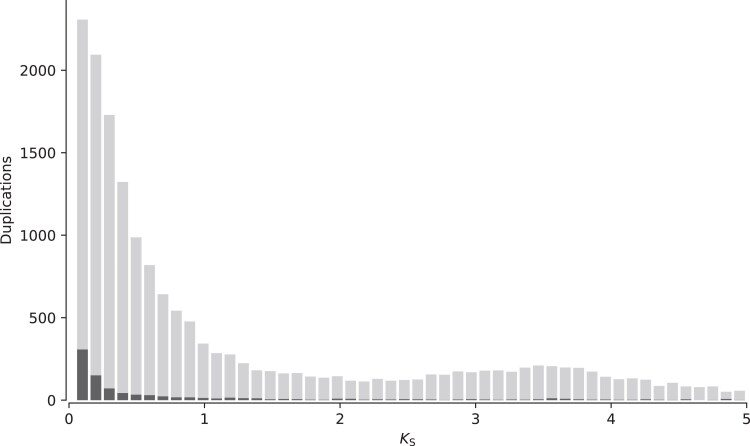
Distribution of synonymous substitutions per synonymous site (*Ks*) for all inferred duplications in the *As. poculata* genome with light gray representing all paralogous gene pairs and black representing anchor gene pairs.

**Fig. 4.**
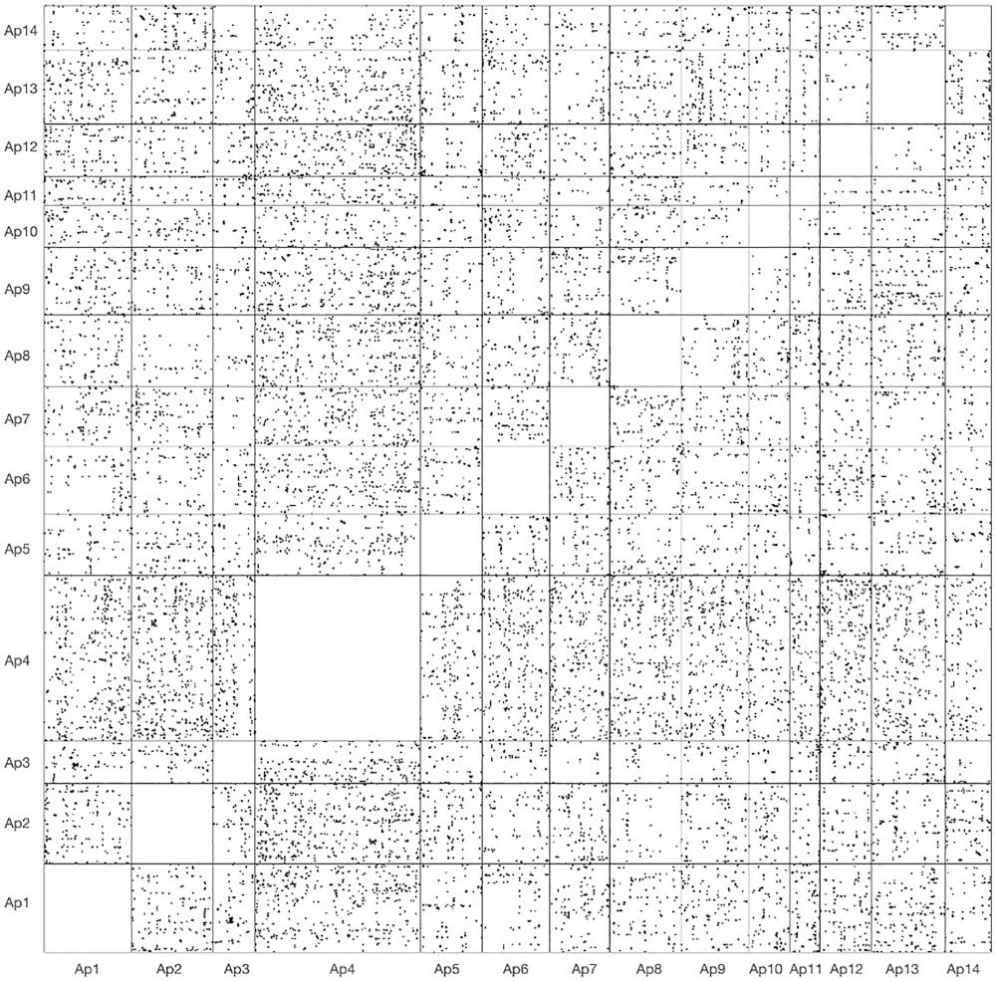
Oxford grid representing pairs of homologous regions detected by SatsumaSynteny across the genome of *As. poculata*. On this grid (not drawn to scale), each point represents a pair of identical or nearly identical 4,096 bp regions. Ap1, Ap2, …, Ap14 represent the 14 chromosome-scale scaffolds in the assembly of the genome of *As. poculata*.

Duplication analysis via MCScanX (Wang *et al*. 2012) revealed 88 syntenic blocks with 853 duplicated genes classified as putatively originating from WGD or segmental duplication (SD; Supplementary Table 4). When relaxing MCScanX parameters by allowing a max gap size of 50 genes within colinear blocks (default gap size is 25), the number of duplications classified as whole genome or segmental increased to 1,005. Detected colinear blocks often involved more than 2 colinear regions, with some involving 3 or even 4 colinear regions. However, a *Ks*-based approach using the tool wgd (Zwaenepoel and Van de Peer 2018) resulted in an exponential decay shape of the distribution of synonymous substitutions per synonymous site, suggesting no signature of a WGD event in *As. poculata* (Fig. 3). While we did detect many anchor pairs (colinear paralogs), the anchor *Ks* distribution also declined exponentially. This was consistent with the failure of Orthodotter to detect large colinear regions in the genome of *As. poculata* (Fig. 4), suggesting that WGD, if it occurred, was too ancient and the genome had subsequently undergone too much rearrangement to leave an obvious colinearity signature. Rather, these results suggest widespread duplications (tandem, proximal, and dispersed) within the *As. poculata* genome, which may have resulted in novel functional gene copies through the process of neofunctionalization (Teshima and Innan 2008).

### Comparative genomics: gene family size and conserved synteny

**Fig. 5.**
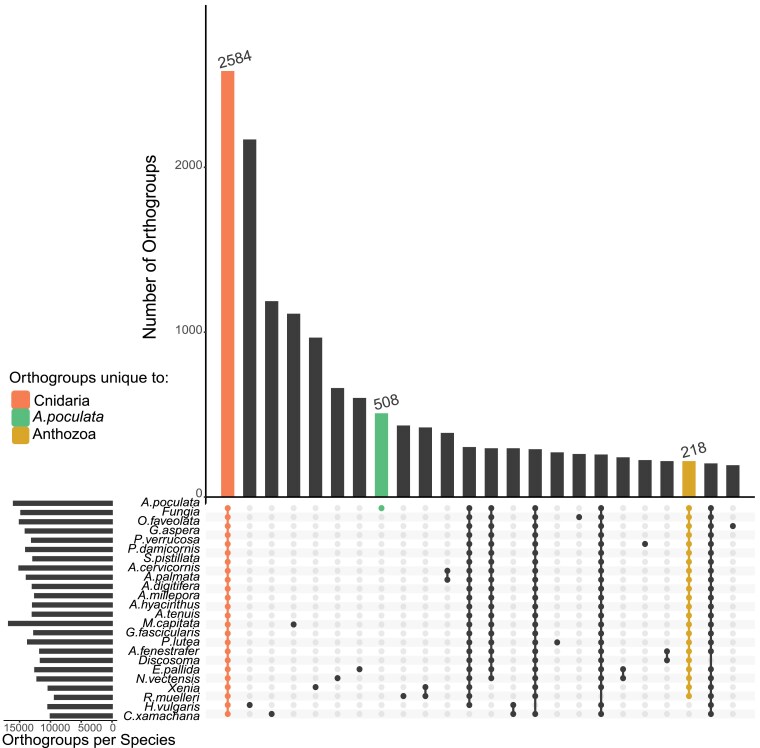
An UpSet plot representing the number of orthogroups containing each species included in the analysis. Each dot represents the presence of a given species in orthogroups, with orthogroups unique to *As. poculata* (green), shared amongst cnidarians (orange), and shared among anthozoans (gold) highlighted.

Using a gene family analysis involving a total of 24 species, we defined a “core” cnidarian genome that consisted of 2,584 gene families shared among all cnidarians present in the analysis (Fig. 5). We found 508 gene families unique to *As. poculata.* Interestingly, only 218 orthogroups were present in all anthozoans included in the analysis. GO enrichment in clusterProfiler (Yu *et al*. 2012) of the gene families unique to *As. poculata* resulted in 143 enriched GO terms (significance threshold of *P* < 0.05 after adjusting for multiple testing; Supplementary Table 5).

**Fig. 6.**
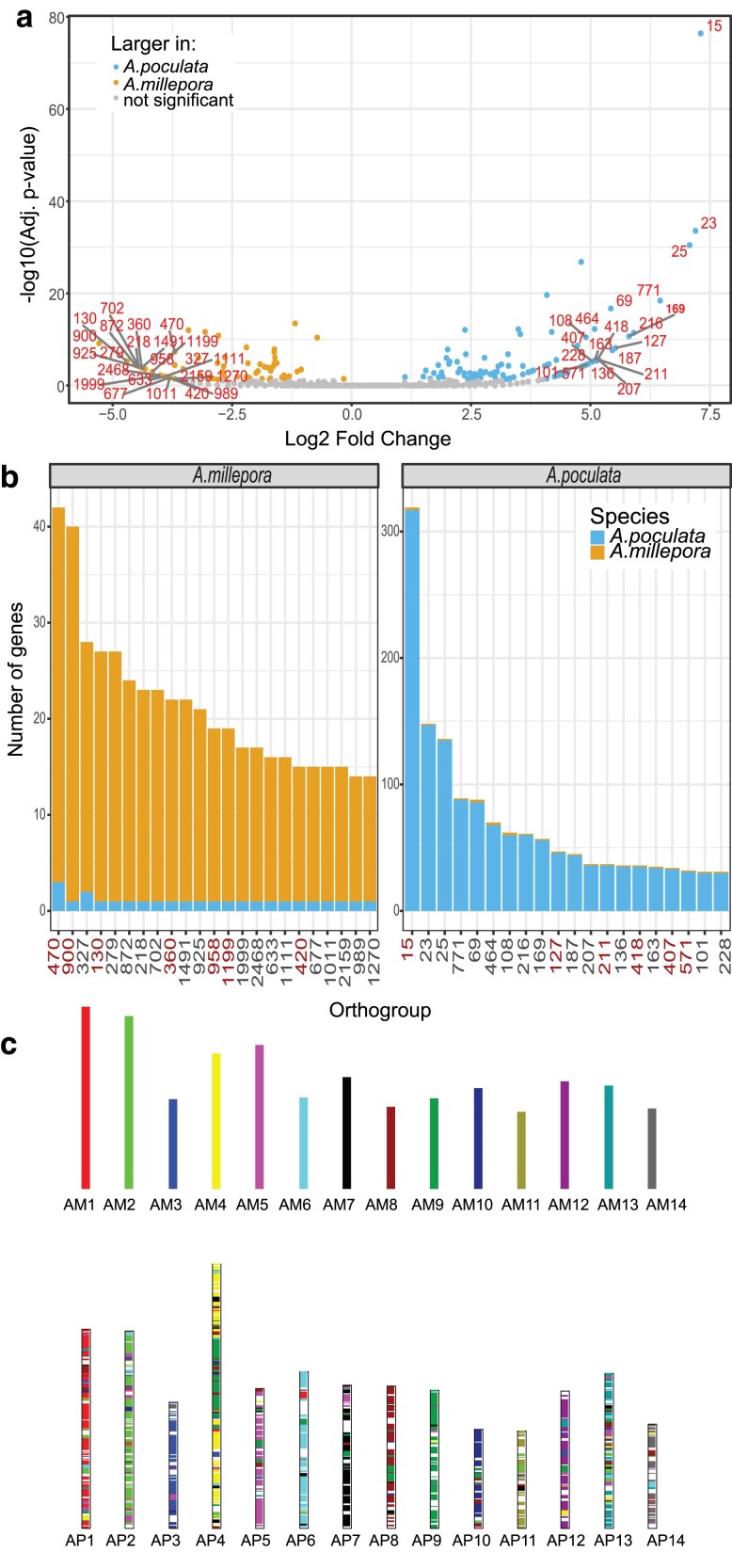
Comparison of *As. poculata* and *Ac. millepora* genomes. a) Volcano plot of gene family size comparison using Fisher's exact test between *As. poculata* and *Ac. millepora*. Points are colored according to whether they are significantly larger in *As. poculata* (blue), significantly larger in *Ac. millepora* (gold), or not significantly different (gray) with adjusted *P* > 0.05. The top 20 [note: equal rank (i.e. equal log_2_ fold change values) orthogroups are all depicted, leading to 23 gene families for *Ac. millepora*] significant gene families for each species are labeled with orthogroup IDs in red. For readability, orthogroups are labeled with leading “OG” and zeros removed from their IDs. b) The number of genes for these top gene families that are significantly larger in *As. poculata* (panel “*A. poculata*”) and *Ac. millepora* (panel “*A. millepora*”). Each bar represents the number of genes in the gene family for *As. poculata* (blue) and *Ac. millepora* (gold). Gene families with putative functions in reproduction, symbiosis, innate immunity, transposition, and quiescence are highlighted in red. c) A bar plot representing the conserved synteny between the genomes of *As. poculata* (AP) and *Ac. millepora* (AM). Each chromosome of *As. poculata* is painted with the color of the *Ac. millepora* chromosome with which there is conserved synteny. White spaces indicate regions where colinear blocks were not detected.

Gene families that were common to *As. poculata* and *Ac. millepora* were assessed for differences in gene numbers using Fisher's exact test following the methods of González-Pech *et al*. (2021). In total, 170 gene families were identified as significantly different in gene numbers between the 2 species (Fig. 6a; Supplementary Table 6; adjusted *P* ≤ 0.05). This included 73 gene families that were significantly larger in *Ac. millepora* and 97 gene families that were significantly larger in *As. poculata.* Gene families larger in *Ac. millepora* that were most different in size compared to *As. poculata* (according to log_2_ fold change) included gene families that putatively encoded for zinc finger CCHC domain-containing proteins, cation channel sperm-associated proteins, RING-box proteins, lectins, and serine/threonine-protein kinases. In contrast, gene families larger in *As. poculata* that were most different in size compared to *Ac. millepora* by log_2_ fold change included orthogroups that putatively encoded for transposable elements, G protein-coupled receptors, zinc finger MYM-type proteins, RNA-directed DNA polymerases, orexin receptors, ATP-dependent DNA helicases, E3 ubiquitin-protein ligases, and histone H3 (Fig. 6b; Supplementary Table 6). GO enrichment analysis resulted in 723 and 635 enriched terms for expanded families in *As. poculata* and *Ac. millepora*, respectively. Enriched terms in *Ac. millepora* expanded gene families included CatSper complex, sperm principal piece, zinc ion binding, and viral latency (Supplementary Table 7), while enriched terms in expanded *As. poculata* families included transposition, G protein-coupled receptor activity, and peptide receptor activity (Supplementary Table 8).

In addition to gene family size analysis, orthologues common to *As. poculata* and *Ac. millepora* were evaluated for conserved synteny. MCScanX analysis of colinearity revealed a high level of conserved gene synteny between *As. poculata* and *Ac. millepora* with 3,719 syntenic blocks identified of at least 5 colinear genes. In total, 56.63% of orthologous genes were present in the colinear blocks between the 2 divergent coral species (Fig. 6c).

### Histology

**Fig. 7.**
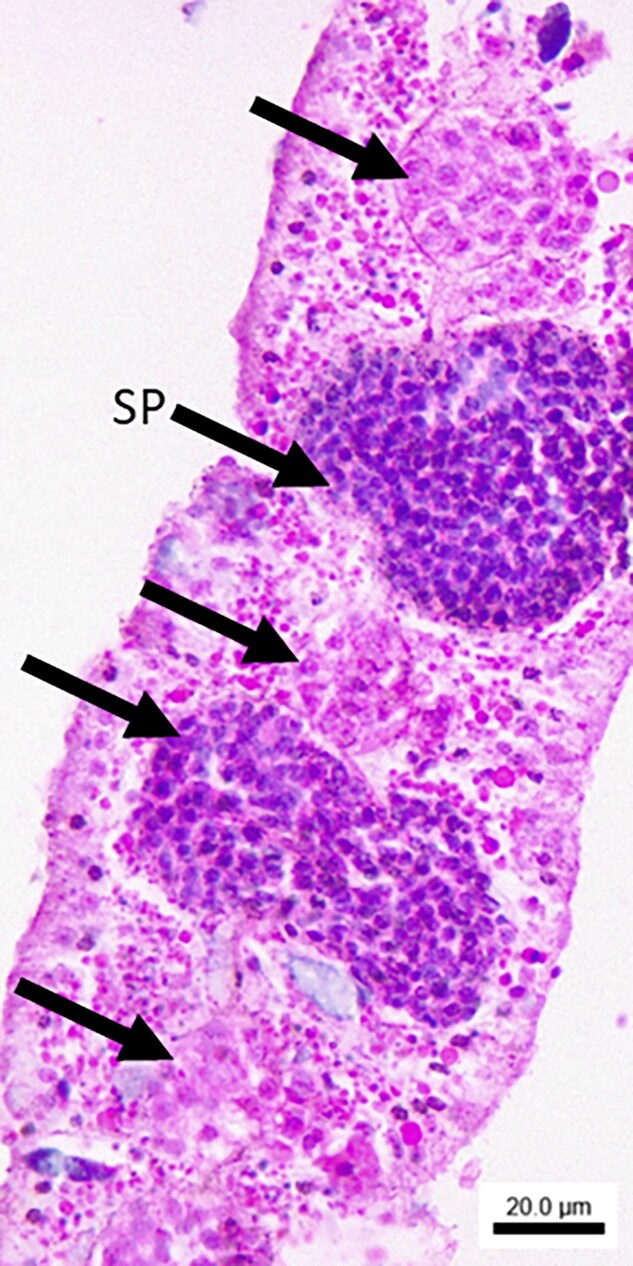
Representative section from the sequenced *As. poculata* colony stained with hematoxylin and eosin. The scale bar = 20 µm. Black arrows point to mesenteries showing developing spermaries (SP).

Examination of the histology slides revealed that the sequenced *As. poculata* colony was male, with developing spermaries in stages II–IV (Szmant *et al.* 1980) in 2 of the tissue sections (Fig. 7).

## Discussion

*Astrangia poculata* has increasingly been used as a model coral system due to its temperature tolerance and flexibility in symbiont state (Jacques *et al*. 1983; Peters *et al*. 1988; Dimond and Carrington 2007; Burmester *et al* 2017; 2018; Sharp *et al*. 2017; Aichelman *et al*. 2019; Chan *et al*. 2021; DiRoberts *et al*. 2021; Wuitchik *et al*. 2021). Because *As. poculata* is facultatively symbiotic, the host and the algal symbiont response to manipulation can be distinguished—a study design that is often impossible in adult tropical corals, many of whom do not occur naturally in an aposymbiotic state. In addition to associating with an algal endosymbiont of the family Symbiodiniaceae, *As. poculata* creates a calcium carbonate skeleton similar to reef-building corals (Hayes and Goreau 1977; Peters *et al*. 1988). However, corals are a diverse group of organisms with respect to their biology and ecology (e.g. habitat, morphology, and response to environmental change) (Chappell 1980; Fabricius *et al*. 2011; Muir *et al.* 2018; Kusumoto *et al*. 2020). Thus, it is important to recognize the differences, as well as the similarities, between *As. poculata* and other corals. Here, we have developed a chromosome-scale genome assembly for *As. poculata*, which has allowed us to characterize some of these differences and similarities between *As. poculata* and other cnidarians. These results provided insight into potential genomic drivers of cnidarian biology, such as *As. poculata's* astounding ability to enter a dormancy state when exposed to extreme cold temperatures, and elucidated the demands placed on sperm during mass spawning events of tropical corals.

### No evidence of ancient whole-genome duplication, but recent duplications are abundant

WGD can generate new genetic material upon which selection or genetic drift may act (Ohno 1970; Holland and Ocampo Daza 2018). A possible WGD event in the most recent common ancestor of the genus *Acropora* was suggested using phylogenomic and comparative genomic techniques (Mao and Satoh 2019). However, it is unknown if such an event may have occurred at other points in the scleractinian lineage where polyploidism is common. Our conservative results from MCScanX indicated that 853 duplicated genes (1.8%) were classified as possibly originating from large-scale duplication events, such as segmental or WGD (Supplementary Table 4). To further identify whether this may indeed be indicative of an ancient WGD event, we examined the distribution of synonymous substitutions per synonymous site (*Ks*). Under a model of a constant rate of duplication and loss, there should be an exponential decay shape to a *Ks* distribution, which is the shape we find in the distribution for *As. poculata* (Fig. 3). In contrast, when a WGD event has occurred, it leaves a signature peak in the *Ks* distribution (Zwaenepoel and Van de Peer 2018). Additionally, we examined the *Ks* distribution of the anchor pairs (paralogs located on colinear duplicated segments). However, many anchors detected represented small *Ks* values (0–0.1) and also followed an exponentially decaying shape. Similarly, a search for microsynteny using SatsumaSynteny followed with colinearity detection using Orthodotter did not reveal abundant pairs of colinear regions (Fig. 4). These results suggest that while large-scale SDs may be present in the *As. poculata* genome, we are unable to detect a strong signature of an ancient WGD event. Because these approaches may not be able to detect very ancient duplications, a phylogenomic approach (Zwaenepoel and Van de Peer 2018) will be required to further test for an ancient WGD event in Scleractinia as additional high-quality genome assemblies representative of each taxonomic group across the cnidarian phylogeny become available.

While there was no evidence of an ancient WGD event in *As. poculata*, we found a high level of large-scale SDs, tandem duplications, and putatively transposon-derived gene models (Table 1). This may explain the higher gene density of *As. poculata* (97.9 genes/Mb; Table 1) relative to the complex coral, *Ac. millepora* (63.4 genes/Mb). The gene density in *As. poculata* is in-line with those of other Robusta corals (*Pocillipora cf effusa* and *Pocillopora damicornis*) where pervasive tandem duplications were also detected (Noel *et al*. 2023).

### Genome content of temperate *As. poculata* vs tropical *Ac. millepora*

Orthologue analysis resulted in 508 orthogroups unique to *As. poculata* (Fig. 5). These gene families were found to be enriched in 143 GO terms, including terms involving ubiquitin E3 ligases, regulation of transcription and gene expression, G protein-coupled receptor activity, and transposition (Supplementary Table 5). To further investigate the unique genomic content of *As. poculata*, we selected one tropical coral to include in a more detailed comparison. The *As. poculata* genome assembly is among the most complete and contiguous coral genomes to date. Many of the other currently available cnidarian genome assemblies remain considerably more fragmented. For this reason, we limited our subsequent genomic analyses to comparisons between *As. poculata* and *Ac. millepora*. *Acropora millepora* has a chromosome-scale genome assembly (Fuller *et al*. 2020), and contrasts with *As. poculata* in its ecology and evolutionary history. While *As. poculata* is a facultatively symbiotic temperate coral of the robust clade, *Ac. millepora* is an obligately symbiotic tropical coral of the complex clade.

### Conserved micro- and macrosynteny between complex and robust clades

Synteny analysis between *As. poculata* and *Ac. millepora* revealed considerable conserved colinearity (56.63%; Fig. 6c) despite ∼415 Mya of divergence of the 2 clades, Robusta (*As. poculata*) and Complexa (*Ac. millepora*) (Stolarski *et al*. 2011). This surprisingly high level of colinearity is in line with previous work comparing other complex and robust coral species. Ying *et al*. (2018) found that the extent of conserved gene order within Scleractinia, regardless of clade, was relatively high compared to the level of conserved synteny between sea anemones *Exaiptasia* and *Nematostella* in the order Actinaria. Our results further lend support to this conclusion of consistently high conserved gene order across scleractinians.

### Differential gene family expansions related to innate immunity and symbiosis

While gene order analysis highlighted similarities between *As. poculata* and *Ac. millepora*, gene family size comparisons revealed differences (Fig. 6a and b; Supplementary Table 6). Gene family expansions are often observed during adaptation in corals (van Oppen and Medina 2020). Notably, of the gene families expanded in *Ac. millepora* relative to *As. poculata*, the gene family with the largest log_2_ fold change contained several copies of zinc finger CCHC domain-containing protein 3 (orthogroup OG0000470; Fig. 6a and b; Supplementary Table 6), which plays a role in the innate immune response to viruses (Lian *et al*. 2018). Defense against viruses is likely important in *Ac. millepora*, as previous work has identified massive viral outbreaks in this species (Correa *et al*. 2016). Further, obligately symbiotic cnidarians have a more advanced innate immunity repertoire relative to nonsymbiotic relatives, possibly driven by the dynamic and constant interaction with the algal endosymbiont (Shinzato *et al*. 2011; Voolstra *et al*. 2017; Cunning *et al*. 2018; Shumaker *et al*. 2019; van Oppen and Medina 2020). These previous findings relied on comparisons to more distantly related, nonsymbiotic anthozoans. However, here we determine that this holds with a comparison to a more closely related facultatively symbiotic coral, as opposed to a noncoral anthozoan relative.

The molecular elements governing the uptake, maintenance, and breakdown of symbiosis in corals still remain largely unclear. Previous work does indicate that features of the innate immune system of the host, notably lectins, play an important role (Kvennefors *et al.* 2008; Fransolet *et al.* 2012; Zhou *et al*. 2018; Hu *et al.* 2020; Takeuchi *et al*. 2021). Lectins are pattern-recognition proteins that bind to carbohydrates (Goldstein *et al.* 1980). Within the top 15 orthogroups significantly larger in *Ac. millepora* relative to *As. poculata* were gene families related to innate immunity that have previously been implicated in establishing symbiotic associations in corals, including C-type lectins and macrophage mannose receptors that mediate endocytosis of glycoproteins (orthogroups OG0000360, OG0000958, and OG0001199; Fig. 6a and b; Supplementary Table 6). Kvennefors *et al*. (2008) isolated a mannose-binding lectin in *Ac. millepora* and demonstrated its affinity to binding to both pathogens and algal dinoflagellates of the family Symbiodiniaceae. Since then, comparative genomics and single-cell RNA sequencing have further emphasized the role of lectins in the cnidarian-algal symbiosis in additional species (Cunning *et al*. 2018; Hu *et al*. 2020). Here, we were able to compare 2 scleractinian corals, one facultatively symbiotic and the other obligately symbiotic. We found that pattern-recognition proteins (e.g. lectins) are expanded in *Ac. millepora* relative to *As. poculata*. Future study characterizing the potential differences in the establishment and maintenance of symbiosis between obligately vs facultatively symbiotic corals is warranted, and the results here highlight lectin-related gene families as excellent targets.

### Sexual reproduction in mass spawning tropical corals

In addition to innate immunity, top gene families larger in *Ac. millepora* compared to *As. poculata* included those related to sexual reproduction (Fig. 6a and b; Supplementary Table 6), with functions involved in sperm cell hyperactivation (cation channel sperm-associated protein subunit epsilon; orthogroup OG0000900) and meiosis (RING-box protein 1; orthogroup OG0000130). Further, significantly enriched GO terms in the *Ac. millepora* expanded gene families included CatSper complex (GO:0036128), sperm principal piece (GO:0097228), and sperm flagellum (GO:0036126; Supplementary Table 7). These findings are interesting because of the difference in reproductive modes between *Ac. millepora* and *As. poculata*. *Astrangia poculata* is a gonochoric species that reproduces via broadcast spawning wherein gametes are released into the water column prior to fertilization (Szmant *et al.* 1980). Spawning occurs annually from August to September based on the seasonal maximum temperature, with a second cycle sometimes observed in October or November (Peters *et al*. 1988). *Astrangia poculata* sperm are unlikely to have to compete with many other corals for the fertilization of eggs because there are only a few other temperate coral species. We were able to confirm that the sequenced *As. poculata* colony was male as indicated by the presence of spermaries (Fig. 7). In contrast, *Ac. millepora* is a hermaphroditic broadcast spawning species that reproduces during “mass spawning” events synchronized with the occurrence of the full moon (Kaniewska *et al.* 2015). During these annual spawning events, *Ac. millepora* releases gametes simultaneously with over 100 other coral species, as well as hundreds of other invertebrates over the course of only a few nights (Babcock *et al*. 1986; Harrison 2011). This creates competition between gametes, as well as the opportunity for interspecific hybridization. Expansion of these reproduction-related gene families has the potential to soften or maintain species boundaries and would be great targets for future studies examining adaptation to mass spawning in corals.

### Genome plasticity in *As. poculata*: transposition and epigenetics

Gene families that were larger in *As. poculata* relative to *Ac. millepora* included families of transposable elements (Fig. 6a and b; Supplementary Table 6; orthogroups OG0000015 and OG0000418). Further, significantly enriched GO terms in the *As. poculata* expanded gene families included transposition (GO:0032196) and DNA-mediated transposition (GO:0006313; Supplementary Table 8). Retrotransposition contributed to gene family expansion in Symbiodiniaceae (Lin *et al*. 2015; González-Pech *et al*. 2021) and, based on our results, may also play a role in the host *As. poculata*. Further, transposable elements promote adaptation and drive genome plasticity in many species, including bacteria, fungi, plants, and animals (Bennett 2004; Feschotte and Pritham 2007; Leitch and Leitch 2008; Mat Razali *et al.* 2019; Yuan *et al*. 2021). Epigenetic modification is involved in host genome regulation of transposable elements (Matzke *et al.* 2000). Interestingly, we also found that a family of histone H3 proteins was expanded in *As. poculata* relative to *Ac. millepora* (orthogroup OG0000255; Fig. 6a and b; Supplementary Table 6). In eukaryotes, histone H3 is one of the core histone proteins involved in structuring chromatin (Kornberg 1977; McGhee and Felsenfeld 1980). The sequence variants, as well as different modification states of histone H3, are thought to influence gene regulation (Sarma and Reinberg 2005; Hake *et al*. 2006; Kouzarides 2007; Loyola and Almouzni 2007; Maehara *et al*. 2015; Jiang and Berger 2017; Klemm *et al.* 2019). In plants, histone H3 plays a role in development and abiotic stress (Yuan *et al.* 2013; Otero *et al.* 2014). These results suggest that transposable elements and epigenetic modification may play an important role in the plasticity of *As. poculata*.

### Winter quiescence in *As. poculata*

Among the top gene families expanded in *As. poculata* was a family of G-coupled protein receptors, including RYamide receptors, orexin receptors, and neuropeptide SIFamide receptors (orthogroup OG0000211; Fig. 6a and b; Supplementary Table 6). Expanded *As. poculata* gene families were also enriched for G protein-coupled receptor activity (GO:0004930; Supplementary Table 8). In *Drosophila*, RYamide receptors are possibly associated with feeding suppression (Ida *et al*. 2011), while neuropeptide SIFamide receptors have been associated with the promotion of sleep (Park *et al.* 2014). Similarly, orexin receptors are known to regulate circadian sleep/wake cycles in mammals (Chemelli *et al*. 1999). The expansion of this gene family in *As. poculata* relative to *Ac. millepora* may explain *As. poculata*'s ability to enter a dormant state when exposed to near-freezing temperatures. During winter months in the intertidal and subtidal regions at the northernmost edges of the species' range, *As. poculata* enters this dormancy, referred to as “winter quiescence,” when water temperatures plummet to below 6°C (Grace 2017). During this state, polyps are retracted and oral discs are puffed out, while feeding is reduced or ceased entirely (Jacques *et al*. 1983; Grace 2017; Supplementary Fig. 1). This finding has relevance to warmer waters, e.g. some Mediterranean anthozoans enter a similar “summer dormancy” state, including corals (Coma *et al.* 2000; Caroselli *et al.* 2015). Overall, these results indicate that gene family expansions may have contributed to the adaption of *As. poculata* to the high variance in environmental conditions that this species experiences temporally and spatially across its range.

## Conclusion

In this study, we present the first chromosome-scale assembly of the facultatively symbiotic, temperate coral *As. poculata*. Our contribution of a high-quality genome resource for *As. poculata* advances the use of this species as an experimental model and lays the groundwork for numerous future studies, as *As. poculata* is an important emerging model for coral health (Neff 2020). Further, comparison of the *As. poculata* genome to the tropical, obligately symbiotic coral *Ac. millepora* uncovered potential genomic drivers of unique features of not only *As. poculata*, but of *Ac. millepora*, as well. Taken together, these results have generated genomic targets for future study of adaptation in these species and emphasize the power of comparative genomics to reveal novel insights into the biology of corals.

## Supplementary Material

jkaf033_Supplementary_Data

## Data Availability

All raw sequence data and the genome assembly have been submitted to NCBI under BioProject PRJNA1123198. The genome assembly and annotation files are also available at https://zenodo.org/records/14110456. Supplemental files are available at https://zenodo.org/records/14226509. The scripts associated with the data analysis presented here are available at https://github.com/kate-stankiewicz/apoculata_genome_assembly. Supplemental material available at G3 online.
