# Supplementary material for: Genomic comparison of the temperate coral Astrangia poculata with tropical corals yields insights into winter quiescence, innate immunity, and sexual reproduction
Source: G3 (Bethesda). 2025 Feb 18;15(4):jkaf033. doi: 10.1093/g3journal/jkaf033 (PMC12005167; doi:10.1093/g3journal/jkaf033)
Supplement: jkaf033_Supplementary_Data [file jkaf033_supplementary_data.zip › Astrangia_poculata_supplement_5mar25.docx]

**Supplementary Figure Captions and Tables**

Supplementary Figure S**1**: Photographs of *A. poculata* transitioning into quiescence from 9°C (pre-dormancy state) to 5°C (transitionary state; retracting tentacles) to 2°C (dormant; tentacles fully retracted, oral discs puffed out).

Supplementary Table S**1:** Summary of assembly statistics across assemblers. For each assembly strategy, information represents pre-purging, polishing, and scaffolding steps. Assembler references: ^1^ Vaser and Šikić (2019); ^2^Kolmogorov et al. (2019); ^3^Koren et al. (2017); ^4^Ruan and Li (2020).

| **Assembler** | **Size (Mb)** | **Number of Contigs** | **N50** | **BUSCO singles (%)** | **BUSCO duplicated (%)** |
| --- | --- | --- | --- | --- | --- |
| Raven v1.8.3^1^ | 369 | 3,296 | 145 kb | 74.4 | 0.6 |
| Flye v2.9^2^ | 765 | 9,877 | 146 kb | 70.3 | 21.0 |
| Canu v2.2^3^ | 594 | 8,508 | 96 kb | 67.0 | 16.6 |
| wtdbg2 v2.5^4^ | 475 | 4,417 | 443 kb | 80.3 | 1.2 |

Supplementary Table S**2**: Summary of the 24 species included in the ortholog analysis. Scleractinian corals are classified according to whether they fall into the ‘Robusta’ or ‘Complexa’ clade. Species are ordered according to phylogeny generated from OrthoFinder results (see Figure 5 in the main text).

| Genus | Species | Clade | Source | Accession | Citation |
| --- | --- | --- | --- | --- | --- |
| *Astrangia* | *poculata* | Robusta | This study | This study | This study |
| *Fungia* | spp. | Robusta | reefgenomics | ffun_1.0.proteins.fasta | Ying et al., 2018 |
| *Orbicella* | *faveolata* | Robusta | zenodo | Orbicella_faveolata_gen_17.proteins.fa | Prada et al. 2016 |
| *Goniastrea* | *aspera* | Robusta | reefgenomics | gasp_1.0.proteins.fast | Ying et al., 2018 |
| *Pocillopora* | *verrucosa* | Robusta | reefgenomics | Pver_proteins_names_v1.0.faa | Buitrago-López et al., 2020 |
| *Pocillopora* | *damicornis* | Robusta | reefgenomics | pdam_proteins.fasta | Cunning et al., 2018 |
| *Stylophora* | *pistillata* | Robusta | reefgenomics | Spis.genome.annotation.pep.longest | Voolstra et al. 2017 |
| *Acropora* | *cervicornis* | Complexa | zenodo | Acropora_cervicornis.proteins.fa | Locatelli et al., 2024 |
| *Acropora* | *palmata* | Complexa | zenodo | Apalm_assembly_v3.1_200911.protein.fasta | Locatelli et al., 2024 |
| *Acropora* | *digitifera* | Complexa | NCBI | GCF_000222465.1 | Shinzato et al., 2011 |
| *Acropora* | *millepora* | Complexa | przeworskilab | Amil.all.maker.proteins.fasta | Fuller et al., 2020 |
| *Acropora* | *hyacinthus* | Complexa | github | Ahyacinthus.proteins.fasta | López-Nandam et al., 2023 |
| *Acropora* | *tenuis* | Complexa | reefgenomics | aten_0.11.maker_post_001.proteins.fasta | Cooke et al., 2020 |
| *Montipora* | *capitata* | Complexa | cyanophora.rutgers | Mcap.protein.fa | Shumaker et al., 2019 |
| *Galaxea* | *fascicularis* | Complexa | reefgenomics | gfas_1.0.proteins.fasta | Ying et al., 2018 |
| *Porites* | *lutea* | Complexa | reefgenomics | plut2v1.1.proteins.fasta | Robbins et al., 2019 |
| *Amplexidiscus* | *fenestrafer* | NA | reefgenomics | afen.prot.fa | Wang et al. 2017 |
| *Discosoma* | spp. | NA | reefgenomics | dspp.prot.fa | Wang et al. 2017 |
| *Exaiptasia* | *pallida* | NA | reefgenomics | aiptasia_genome.proteins.fa | Baumgarten et al., 2015 |
| *Nematostella* | *vectensis* | NA | NCBI | GCF_932526225.1 | Fletcher et al., 2023 |
| *Xenia* | spp. | NA | carnegiescience | xenSp1.proteins.fa | Hu et al. 2020 |
| *Renilla* | *muelleri* | NA | reefgenomics | renilla_predicted_proteins.fa | Jiang et al. 2019 |
| *Hydra* | *vulgaris* | NA | NHGRI | HVAEP.predictedprot.fa | Simakov et al., 2022 |
| *Cassiopea* | *xamachana* | NA | JGI | Casxa1_GeneCatalog_proteins_20200306.aa.fasta | Ohdera et al. 2019 |

Supplementary Table S**3**: Scaffold-level summary statistics for the *A. poculata* genome assembly.

| **Chromosome-level Scaffolds** | |
| --- | --- |
| **Scaffold ID** | **Length** |
| Ap1 | 42928715 |
| Ap2 | 42634786 |
| Ap3 | 27282911 |
| Ap4 | 58409227 |
| Ap5 | 30212294 |
| Ap6 | 33861578 |
| Ap7 | 31044737 |
| Ap8 | 30683508 |
| Ap9 | 29863146 |
| Ap10 | 21532011 |
| Ap11 | 21106950 |
| Ap12 | 29602567 |
| Ap13 | 33534404 |
| Ap14 | 22725890 |
| **Unplaced Scaffolds** | |
| **Metric** | **Value** |
| sum | 2248290 |
| n | 473 |
| ave | 4753.26 |
| largest | 66459 |
| N50 | 7563 |

Supplementary Table S**4:** Summary statics of duplication classification in the *A. poculata* genome with both default MCScanX parameters and with relaxed parameters that allow a gap of up to 50 genes.

| Type of duplication | Number with default parameters | Number with relaxed parameters |
| --- | --- | --- |
| Singleton | 11,063 | 11,063 |
| Dispersed | 20,794 | 20,702 |
| Proximal | 6,248 | 6,223 |
| Tandem | 8,198 | 8,163 |
| WGD or segmental | 853 | 1,005 |

Supplementary Table S**5**: GO annotations for the 143 terms enriched in the gene families unique to *A. poculata* relative to all other cnidarians included in this analysis (see Supplementary Table S2).

Table is included as a separate file due to its large size and is available on Zenodo at: https://zenodo.org/records/14226509

Supplementary Table S**6**: The gene annotations for all genes included in the gene families that were significantly different in size between *A. poculata* and *A. millepora*.

Table is included as a separate file due to its large size and is available on Zenodo at: https://zenodo.org/records/14226509

Supplementary Table S**7**: GO annotations for significantly enriched terms in the 73 gene families that were significantly larger in *A. millepora* relative to *A. poculata*.

Table is included as a separate file due to its large size and is available on Zenodo at: https://zenodo.org/records/14226509

Supplementary Table S**8**: GO annotations for significantly enriched terms in the 97 gene families that were significantly larger in *A. poculata* relative to *A. millepora*.

Table is included as a separate file due to its large size and is available on Zenodo at: https://zenodo.org/records/14226509

References for Supplementary Table S**2**:

Baumgarten, S., Simakov, O., Esherick, L. Y., Liew, Y. J., Lehnert, E. M., Michell, C. T., ... & Voolstra, C. R. (2015). The genome of *Aiptasia*, a sea anemone model for coral symbiosis. *Proceedings of the National Academy of Sciences*, *112*(38), 11893-11898.

Buitrago-López, C., Mariappan, K. G., Cárdenas, A., Gegner, H. M., & Voolstra, C. R. (2020). The genome of the cauliflower coral *Pocillopora verrucosa*. *Genome biology and evolution*, *12*(10), 1911-1917.

Chapman, J. A., Kirkness, E. F., Simakov, O., Hampson, S. E., Mitros, T., Weinmaier, T., ... & Steele, R. E. (2010). The dynamic genome of *Hydra*. *Nature*, *464*(7288), 592-596.

Cooke, I., Ying, H., Forêt, S., Bongaerts, P., Strugnell, J. M., Simakov, O., Zhang, J., Field, M. A., Rodriguez-Lanetty, M., Bell, S. C., Bourne, D. G., van Oppen, M. J. H., Ragan, M. A., & Miller, D. J. (2020). Genomic signatures in the coral holobiont reveal host adaptations driven by Holocene climate change and reef specific symbionts. *Science Advances*. <https://doi.org/10.1126/sciadv.abc6318>

Fletcher C, Pereira da Conceicoa L, Natural History Museum Genome Acquisition Lab et al. (2023). The genome sequence of the starlet sea anemone, *Nematostella vectensis* (Stephenson, 1935) [version 1; peer review: 1 approved, 1 approved with reservations, 1 not approved]. *Wellcome Open Res 2023, 8:79* https://doi.org/10.12688/wellcomeopenres.18991.1

Jiang, J. B., Quattrini, A. M., Francis, W. R., Ryan, J. F., Rodríguez, E., & McFadden, C. S. (2019). A hybrid de novo assembly of the sea pansy (*Renilla muelleri*) genome. *GigaScience*, *8*(4), giz026.

López-Nandam EH, Albright R, Hanson EA, Sheets EA, Palumbi SR. Mutations in coral soma and sperm imply lifelong stem cell renewal and cell lineage selection. Proc Biol Sci. 2023 Jan 25;290(1991):20221766. doi: 10.1098/rspb.2022.1766. Epub 2023 Jan 18. PMID: 36651044; PMCID: PMC9846893.

Ohdera, A., Ames, C. L., Dikow, R. B., Kayal, E., Chiodin, M., Busby, B., ... & Ryan, J. F. (2019). Box, stalked, and upside-down? Draft genomes from diverse jellyfish (Cnidaria, Acraspeda) lineages: *Alatina alata* (Cubozoa), *Calvadosia cruxmelitensis* (Staurozoa), and *Cassiopea xamachana* (Scyphozoa). *GigaScience*, *8*(7), giz069.

Prada, C., Hanna, B., Budd, A. F., Woodley, C. M., Schmutz, J., Grimwood, J., ... & Medina, M. (2016). Empty niches after extinctions increase population sizes of modern corals. *Current Biology*, *26*(23), 3190-3194. doi: 10.1016/j.cub.2016.09.039

Putnam, N. H., Srivastava, M., Hellsten, U., Dirks, B., Chapman, J., Salamov, A., ... & Rokhsar, D. S. (2007). Sea anemone genome reveals ancestral eumetazoan gene repertoire and genomic organization. *Science*, *317*(5834), 86-94. doi: 10.1126/science.1139158

Robbins, S. J., Singleton, C. M., Chan, C. X., Messer, L. F., Geers, A. U., Ying, H., ... & Bourne, D. G. (2019). A genomic view of the reef-building coral *Porites lutea* and its microbial symbionts. *Nature Microbiology*, *4*(12), 2090-2100. doi: 10.1038/s41564-019-0532-4

Simakov, O., Bredeson, J., Berkoff, K., Marletaz, F., Mitros, T., Schultz, D. T., O’Connell, B. L., Dear, P., Martinez, D. E., Steele, R. E., Green, R. E., David, C. N., & Rokhsar, D. S. (2022). Deeply conserved synteny and the evolution of metazoan chromosomes. Science Advances, 8(5), eabi5884.

Wang, X., Liew, Y. J., Li, Y., Zoccola, D., Tambutte, S., & Aranda, M. (2017). Draft genomes of the corallimorpharians *Amplexidiscus fenestrafer* and *Discosoma* sp. *Molecular Ecology Resources*, *17*(6), e187-e195.
