# Supplementary figures and images for: Genomic comparison of the temperate coral Astrangia poculata with tropical corals yields insights into winter quiescence, innate immunity, and sexual reproduction
Source: G3 (Bethesda). 2025 Feb 18;15(4):jkaf033. doi: 10.1093/g3journal/jkaf033 (PMC12005167; doi:10.1093/g3journal/jkaf033)

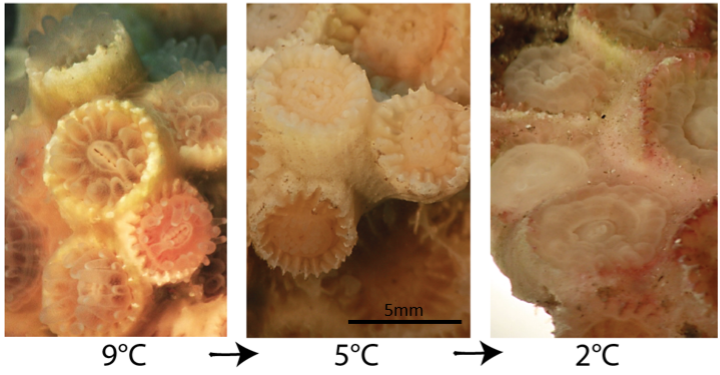

Supplement: jkaf033_Supplementary_Data [file jkaf033_supplementary_data.zip › Supplementary_Figure_S1_G3-2025-405694.png]
